# Keratin 19 marks poor differentiation and a more aggressive behaviour in canine and human hepatocellular tumours

**DOI:** 10.1186/1476-5926-9-4

**Published:** 2010-02-18

**Authors:** Renee GHM van Sprundel, Ted SGAM van den Ingh, Valeer J Desmet, Azeam Katoonizadeh, Louis C Penning, Jan Rothuizen, Tania Roskams, Bart Spee

**Affiliations:** 1 Department of Clinical Sciences of Companion Animals, Faculty of Veterinary medicine, Utrecht University, Utrecht, The Netherlands; 2 TCCI Consultancy BV, Utrecht, The Netherlands; 3 Department of Morphology and Molecular Pathology, University Hospitals Leuven, Leuven, Belgium

## Abstract

**Background:**

The expression of Keratin 19 (K19) was reported in a subset of hepatocellular carcinomas (HCCs). K19 positive HCCs are associated with an increased malignancy compared to K19 negative HCCs. No suitable mouse models exist for this subtype of HCC, nor is the incidence of K19 expression in hepatocellular neoplasia in model animals known. Therefore, we compared the occurrence and tumour behaviour of K19 positive hepatocellular neoplasias in dog and man.

**Results:**

The expression of hepatocellular differentiation (HepPar-1), biliary/progenitor cell (K7, K19), and malignancy (glypican-3) markers was semi-quantitatively assessed by immunohistochemistry. The histological grade of tumour differentiation was determined according to a modified classification of Edmondson and Steiner; the staging included intrahepatic, lymph node or distant metastases. Four of the 34 canine hepatocellular neoplasias showed K19 positivity (12%), of which two co-expressed K7. K19 positive tumours did not express HepPar-1, despite the histological evidence of a hepatocellular origin. Like in human HCC, all K19 positive hepatocellular neoplasias were glypican-3 positive and histologically poorly differentiated and revealed intra- or extrahepatic metastases whereas K19 negative hepatocellular neoplasias did not.

**Conclusions:**

K19 positive hepatocellular neoplasias are highly comparable to man and occur in 12% of canine hepatocellular tumours and are associated with a poorly differentiated histology and aggressive tumour behaviour.

## Background

Hepatic progenitor cells (HPCs) are activated in the majority of liver diseases and are a potential cell of origin for hepatocellular carcinoma (HCC) [1,2]. HCC is a neoplasm of increasing incidence worldwide and is the fifth leading cause of death on a worldwide basis in man [3,4]. Although remarkable advances in surgical and imaging modalities have improved the prognosis of HCC patients [5], the high incidence of intrahepatic recurrence remains a major challenge in HCC therapy [6,7]. In man the only potentially curative modality for HCC is surgical resection (including whole organ transplantation), yet recurrence rates are high and the long-term survival is poor [8]. An additional dilemma is the limited availability of healthy donor livers. Thus, the ability to predict individual recurrence risk and subsequently prognosis would help guide surgical and chemotherapeutic treatment. As the understanding of hepatocarcinogenesis increases, the innumerable genetic and molecular events that drive the hepatocarcinogenic disease process, including angiogenesis, invasion and metastasis, are being unravelled in the human clinical situation.

Keratin (K) 19 expression is normally found in hepatic progenitor cells (HPCs) and cholangiocytes but not hepatocytes [9-11]. However, several authors report the peculiar expression of K19 in HCC in man [12-15]. These K19 expressing HCCs had a higher rate of recurrence (hazard ratio 12.5) after transplantation [6]. Other studies also linked increased K19 expressions in HCC with a worse prognosis and faster recurrence after surgical treatment [14,16-18]. Others observed a significantly shorter survival in patients with HCCs expressing K19 without any treatment [15]. Furthermore, one recent report showed that HCCs expressing K19 and K7 have a lower tumour free survival rate after curative resection [13]. Several studies show that a cut-off of five percent K19 positive cells already influences the outcome of the patient [12]. These studies in man validate K19 as a clinically meaningful and prognostically relevant marker for hepatocellular carcinoma.

Other recently described markers include glypican-3 (GPC3) which is an extracellular proteoglycan that is inferred to play an important role in growth control in embryonic mesodermal tissues in which it is selectively expressed [19]. GPC3 is a member of the glypican family of glucosyl-phosphatidylinositol-anchored cell-surface heparin sulfate proteoglycans and is well established as a serologic and immunohistochemical diagnostic tool for hepatocellular carcinomas in man. The presence of GPC3 (mRNA and immuno-histochemistry) is much higher in hepatocellular carcinomas compared to cirrhotic tissue or small focal lesions, indicating that the transition from small premalignant lesions to hepatocellular carcinomas is associated with a sharp increase of GPC3 expression in the majority of cases [20,21].

In view of the similarities in cell biological mechanisms involved in regeneration and tumour development between human liver tumours and liver tumours in small domestic animals, it is conceivable that these acquisitions found in human hepatic tumour pathology may also be true for the canine liver tumours [22]. To this date, no mouse models exist which resemble K19 positive HCCs in man. Therefore clinicopathological prognostic markers including marker expression of K7, K19 (HPC and cholangiocytes), HepPar-1 (hepatocytes) and glypican-3 (malignant HCC) were examined in primary liver tumours of dogs and compared to man. Results indicate a high similarity in histopathology of primary liver tumours between man and dog, emphasizing the use of dogs as possible treatment models.

## Results

### Histological classification of canine primary liver tumours

Liver material of 46 dogs was included in this study (male to female ratio: 0.7). Breeds represented included mixed breed, Flat coated retriever, Airedale terrier, German Sheppard, Alaskan malamute, Pit bull, Maltezer, Cocker spaniel, Appenzeller, Golden retriever and Yorkshire terrier. The age range was six to fourteen years. Microscopical examination (Table 1) classified the 46 primary liver tumours as: four nodular hyperplasia (9%) and 34 hepatocellular tumours (74%). Five hepatic carcinoids (11%) positive for one or more neuro-endocrine differentiation markers (chromogranin-A, neuron-specific enolase, and synaptophysin) and three cholangiocellular carcinomas (7%) were not further analysed in this study. Apart from the neoplastic changes, no additional liver pathology was seen in any of the dogs. Healthy liver tissue was added as a control. Hepatocellular tumours were classified in different groups based on K19 positivity. Hepatocellular groups were subdivided in K19 negative and K19 positive. In retrospect, all groups were compared with the results of histological markers (staging and grading) and the immunohistochemical markers K7, glypican-3, and HepPar-1.

**Table 1:** Overview of the canine histological classification.

Groups	K19 expression	Grading 0 to 3	Staging 0 to 2	K7 expression	Glypican-3 expression	HepPar-1 expression
Normal liver (n = 5)	0%	0	0	0%	0%	100%
Nodular hyperplasia (n = 4)	0%	0	0	0%	0%	100%
Hepatocellular tumour K19 negative (n = 30)	0-5%	1 (n = 24) 2 (n = 6)	0	0% (n = 29) 5% (n = 1)	0%	50-75% (n = 2) 90-100% (n = 28)
Hepatocellular tumour K19 positive (n = 4)	10-90%	3	1 - 2	0% (n = 2) 5% (n = 2)	30-100%	0%

Grouping based on histology and K19 expression in hepatocytes compared with the results of the grading, staging, and clinicopathological markers

### Nodular hyperplasia (n = 4)

**Figure 1:**
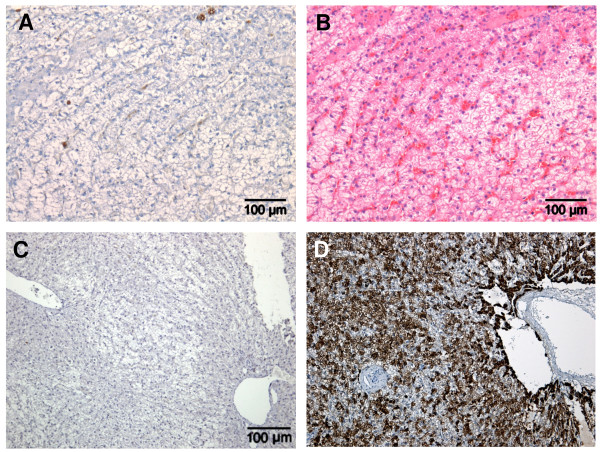
**Examples of canine nodular hyperplasia**. Immunohistochemical staining of K19 negative cells is shown in (A). HE staining, double layered cords of well differentiated hepatocytes are shown in (B). Absence of immunohistochemical staining for glypican-3 is shown in (C). Positive immunohistochemical staining for HepPar-1 is shown in (D).

No K19 expression was observed in the nodular hyperplasia group (Figure 1A). Histologically, lesions consisted of double-layered cords of well-differentiated hepatocytes and slight compression of the surrounding parenchyma. Cells had a similar shape and size, indicating a good uniformity with no cell pleomorphism. No multinucleated hepatocytes were present. There was no mitotic activity and portal areas were present (Figure 1B). All nodular hyperplasias were negative for Glypican-3 (Figure 1C) and strongly positive for HepPar-1 (Figure 1D).

### Hepatocellular tumour K19 negative (n = 30)

**Figure 2:**
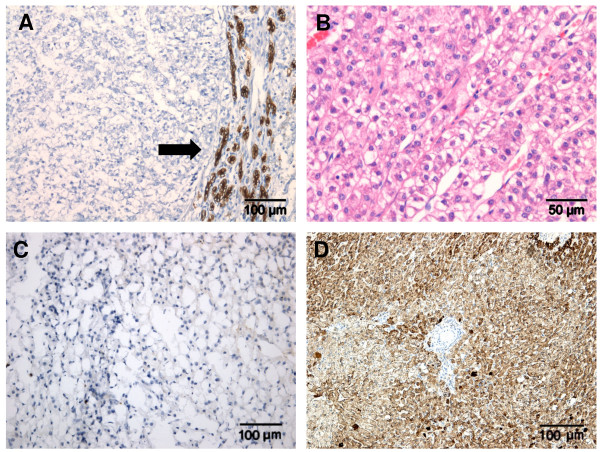
**Examples of canine K19 negative hepatocellular tumours**. Immunohistochemical staining of K19 with a negative tumour field (left) and positive reactive ductular proliferation at the periphery of the tumour (arrow) is shown in (A). HE staining, trabeculae of well-differentiated hepatocytes with a uniform appearance are shown in (B). Absence of immunohistochemical staining for Glypican-3 is shown in (C). Positive immunohistochemical staining for HepPar-1 is shown in (D).

K19 expression in none or less than five percent of the tumour cells was observed in 30 of the 34 hepatocellular tumours (88%) (Figure 2A). Histologically, these tumours formed trabeculae of well differentiated hepatocytes. Cells were uniform in shape and size and with none to little pleomorphism. The nuclei were round and regular with minimal nuclear irregularity; the nucleoli were uniform and sometimes prominent. There were no multinucleated cells and mitotic figures were absent or very rare (Figure 2B). In two cases the tumour cells were not of the same size and were therefore classified as stage two. However the majority of cells were well differentiated and occasionally multinucleated cells could be seen. All tumours were negative for glypican-3 (Figure 2C) and strongly positive for HepPar-1 (Figure 2D).

### Hepatocellular tumour K19 positive (n = 4)

**Figure 3:**
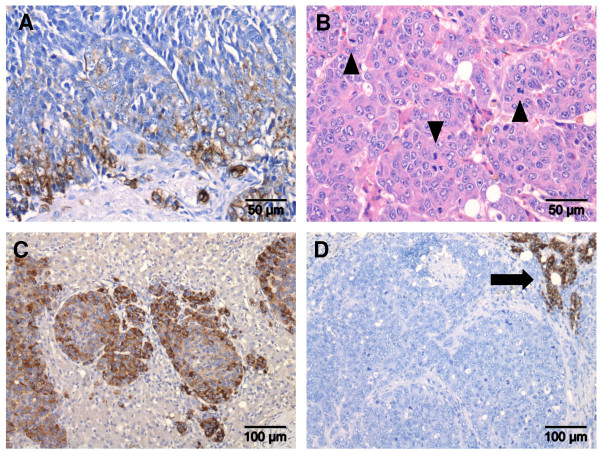
**Examples of canine hepatocellular tumours with high K19 expression**. Immunohistochemical staining of K19 positive cells is shown in (A). HE staining, trabeculae of hepatocytes with cell pleomorphism and multiple mitotic figures (arrowheads) are shown in (B). Immunohistochemical staining of glypican-3 positive cells is shown in (C). Immunohistochemical staining for HepPar-1 with tumour negative area and positive area of surrounding non-neoplastic liver (arrow) is shown in (D).

Keratin 19 expression in 30-90% of the tumour cells was seen in four of the 34 hepatocellular tumours (12%) (Figure 3A). Histologically, these tumours formed irregular trabeculae and were poorly differentiated regarding the cell- and nuclear-morphology. The cells had different shapes and varied in size (anisocytosis). There was much cell pleomorphism and the cell uniformity disappeared. The nuclei were irregular in shape and size (anisokaryosis) and some multinucleated cells could be observed. The nucleoli were very prominent in shape and colour. The mitotic activity was very high (Figure 3B). Tumours were categorized in the most malignant group of the grading system (grade 3) and classified in stage one or two (due to presence of intrahepatic or distant metastasis). The marker glypican-3 was strongly positive (30-100%) for all tumours (Figure 3C) and no HepPar-1 staining was found (Figure 3D).

### K19 positive and negative human hepatocellular tumours (n = 4/group)

**Figure 4:**
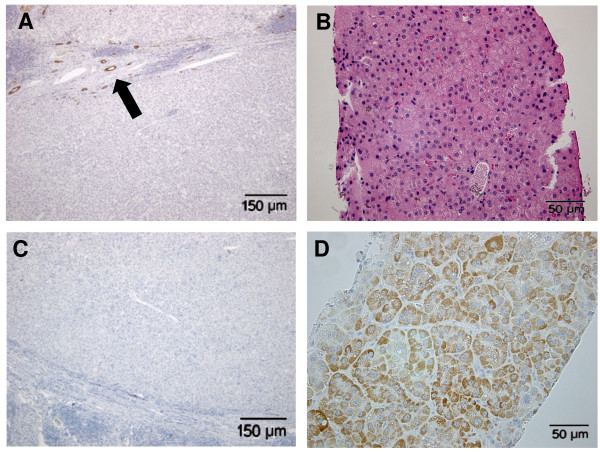
**Examples of K19 negative human hepatocellular tumours**. Immunohistochemical staining of K19 negative cells is shown in (A), positive bile-ducts at the periphery of the tumour indicated by arrow. HE staining, moderately differentiated hepatocytes with trabecular growth pattern is shown in (B), absence of immunohistochemical staining for Glypican-3 is shown in (C). Positive immunohistochemical staining for HepPar-1 is shown in (E).

**Figure 5:**
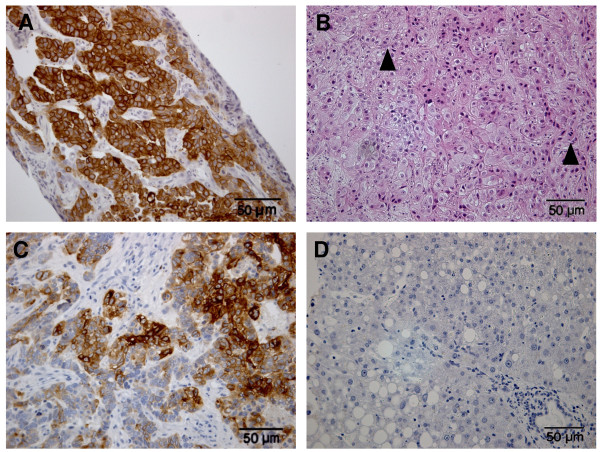
**Examples of K19 positive human hepatocellular tumours**. Immunohistochemical staining of K19 positive cells is shown in (A). HE staining, poorly differentiated HCC with a diffuse growth pattern and multiple mitotic figures (arrowheads) is shown in (B). Immunohistochemical staining for glypican-3 positive cells is shown in (C). Absence of immunohistochemical staining for HepPar-1 is shown in (D).

Eight human hepatocellular neoplasms were selected of which four were K19 negative (Figure 4) and four were K19 positive in 30 to 90 percent of the tumour cells (Figure 5). Histologically, the selected K19 negative tumours were well differentiated and formed trabeculae. Little pleiomorphism was observed and cells were uniform in shape and size. Minimal nuclear irregularity was seen. Occasionally multinucleated cells were seen and mitotic figures were absent or rare (Figure 4B). Keratin negative HCCs were categorized as grade one and classified in stage 0 due to the lack of vascular invasion in these samples or distant metastasis (Table 2). All tumours were negative for glypican-3 (Figure 4C) and strongly positive for HepPar-1 (Figure 4D). Keratin 19 positive tumours histologically had irregular growth patterns and were poorly differentiated. Tumour cells and nuclei were polymorph. The mitotic activity was high (Figure 5B). Tumours were categorized in the most malignant group of the grading system (grade 3) and classified in stage one or two (due to presence of intrahepatic or distant metastasis). The marker glypican-3 was strongly positive (30-100%) for all tumours (Figure 5C) and no HepPar-1 staining was found (Figure 5D).

**Table 2:** Overview of the staging and grading of K19 positive hepatocellular tumours in man.

Groups	K19 expression	Grading 0 to 3	Staging 0 to 2	K7 expression	HepPar-1 expression	Glypican-3 expression
Hepatocellular tumour K19 negative (n = 4)	0%	1	0	0	90-100%	0%
Hepatocellular tumour K19 positive (n = 4)	30-90%	3	1 - 2	100%	0%	30-100%

Grouping based on K19 expression compared with the results of the grading, staging, and clinicopathological markers

### Statistical analysis

Keratin 19 positivity was not found to be linked with age (*P *= 0.17). Keratin 19 positivity was negatively correlated with HepPar-1 staining (*P *= 0.001), and positively correlated with glypican-3 staining (*P *= 0.0001). Keratin 19 positive tumours had significantly more distant metastasis (stage 2) and showed a poorly differentiated histology (grade 3) in comparison with K19 negative tumours (*P *= 0.001 and 0.0002 respectively).

## Discussion

The presence of K19 is a strong and independent predictor of tumour recurrence in man [7,13,14,23,24]. This study investigated the occurrence of K19 negative and positive hepatocellular tumours in dogs and clinicopathological parameters of these tumours and compared these with K19 negative and positive hepatocellular tumours from humans. K19 negative tumours occurred in 88 percent of the canine hepatocellular tumours. Tumours with K19 expression was found in twelve percent of the tumours and were correlated with glypican-3 (marker of malignant change) expression and increased malignancy based on histological grading and staging of the tumours.

The occurrence of K19 positive hepatocellular carcinoma in dogs is twelve percent. In man, several studies estimate the occurrence of the K19 positive phenotype between 9 and 29 percent (median 17 percent) of all hepatocellular carcinomas [12,13,15,25,26]. Recently a study of 417 primary HCCs at the University Hospitals in Leuven, Belgium, showed that 54 were positive for K19 (13 percent, data not shown). The high similarity in occurrence between man and dog confirm the resemblance of K19 positive tumours between species.

The presence of progenitor cell features in a tumour can be explained in two ways: either the cell of origin is a progenitor cell (maturation arrest theory) or alternatively, tumours dedifferentiate and acquire progenitor cell features during carcinogenesis (dedifferentiation theory) [23,27]. When progenitor cells are the cells of origin of a subtype of primary liver tumours, one would expect that the earliest premalignant precursor lesions also would consist of progenitor cells and their progeny. This is indeed the case; 55 percent of small cell dysplastic foci (smaller than 1 mm), the earliest premalignant lesion known to date in humans, consist of progenitor cells and intermediate hepatocytes [28]. This is a very strong argument in favour of the progenitor cell origin of at least part of the HCCs. Large cell 'dysplastic' foci, on the other hand, consists of mature senescent hepatocytes being a result of continuous proliferation in chronic liver diseases and is not the true precursor lesion of HCC. In the veterinary field, little is known about markers of HCC or cholangiocarcinoma with only a few prognostic markers, such as alpha-feto protein (AFP), investigated [29]. Unfortunately the usefulness of AFP as a serum tumour marker is questionable since AFP is only detectable after a significant tumour burden [30].

In the present study, all the canine hepatocellular tumours with K19 expression were categorized in the most malignant group of the grading and staging system which included presence of infiltrative growth, vascular invasion and metastases. These features are linked with a poor prognosis. In contrast, hepatocellular tumours in dogs which do not express K19 have a benign or less malignant character because none of these tumours showed intrahepatic or extrahepatic metastasis and were classified in group one or two of the grading system. However, in the progression of the disease it cannot be excluded that K19 negative tumours will express K19 as time progresses and thereafter become more malignant tumours. It is therefore necessary to follow patients with hepatocellular tumours over time to investigate if these tumours acquire K19 positivity and show an increase in malignancy. Serial biopsies are hard if not impossible to obtain from human livers. In contrast longitudinal studies are ethically much more accepted in dogs.

It is unclear whether the presence of K19 is a mediator or just an epiphenomenon of a more aggressive phenotype. Interestingly, some authors suggest K19 provides tumour cells with a higher metastatic potential by promoting extracellular matrix degradation and/or cell mobility [31,32]. In a murine tumour model Chu et al. established that cells expressing intact keratins had higher *in vitro *mobility and invasiveness [33]. In addition they suggested that intact keratins may act as anchors for specific cell membrane receptors, consequently reducing cell clustering and aiding cell motility. It has been shown that the release of keratin-fragments could contribute to an invasive phenotype [33]. Keratin fragments are released into the blood by malignant epithelial cells by activating proteases which degrade keratins [34-36]. Other authors observed the strong binding of recombinant K19 to laminin, a major protein in all basement membranes, provoking an immune response that damaged the basement membrane [31,32]. These or other mechanisms might contribute to vascular invasion observed in this study, which remains to be proven.

In man, glypican-3 (GPC3) can be an important aid in the morphologically difficult diagnosis between small HCCs and other small focal lesions. The expression of GPC3 in a small focal lesion present in a cirrhotic liver in man is highly indicative of a HCC, irrespective of the percentage of positive cells. The presence of GPC3 (mRNA and immunohistochemistry) is higher in HCCs compared to cirrhotic tissue or small focal lesions, indicating that the transition from small premalignant lesions to HCC is associated with a sharp increase of GPC3 expression in the majority of cases [21,28]. Because GPC3 is over expressed in human hepatocellular carcinoma, this marker is used for hepatocellular tumours in human medicine as a marker for malignant change [37-39]. In this study, all the canine tumours with a K19 expression had 30-100% positivity for glypican-3; all the other hepatocellular tumours were negative for glypican-3. Thus, like K19, expression of glypican-3 seems to be linked with a poor prognosis. Therefore, glypican-3 can be used as a marker for hepatocellular malignancy in dogs.

In this study, no K19/GPC3 positive hepatocellular tumours express the hepatocyte marker HepPar-1. This is consistent with a HPC phenotype of these tumours as HPCs/reactive ductules are also negative for HepPar-1. Another explanation could be that these tumours are dedifferentiated to the point where they do not express HepPar-1 anymore. All K19 expressing hepatocellular tumours which are negative for HepPar-1 are categorized in the highest (most malignant) groups of the grading and the staging system. This suggests a negative correlation between the expression of HepPar-1 and prognosis.

Better characterisation of hepatic tumours by cell surface markers and the use of fluorescence activated cell sorting might in the future contribute to isolation of different tumour cell populations. This will further pave the way for cell-subset-specific gene expression profiling of potential tumour stem cells, other tumour cells and stromal cell populations. In the light of this paradigm, K19 expression in hepatic tumours might correlate with the presence of tumour stem cells deriving from hepatic progenitor cells. If the arising paradigm is verified, a further deepening of our understanding of hepatocellular carcinogenesis is expected. Cell-subset-specific gene expression profiling might indeed uncover specific signalling pathways in tumour stem cells and interactions between tumour stem cells, other tumour cells and stromal cells, which might well be masked in gene expression profiling of the tumour as a whole. Future prognostic modelling will probably encompass molecular markers reflecting the biology and natural history of hepatic tumours [40]. The most interesting perspective is when these markers will also determine the applicability of tailored therapy for which the dog would fit as a highly relevant model.

## Conclusions

K19 positive hepatocellular neoplasias occur in twelve percent of hepatocellular neoplasias and are associated with a poorly differentiated histology and more aggressive tumour behaviour. K19 expression correlates with the expression of glypican-3 and with the disappearance of the hepatocyte marker HepPar-1 and are valuable clinicopathological and prognostic markers in the histopathological diagnosis of hepatocellular tumours in dogs. K19 positive tumours are highly comparable in histology, marker expression, and prevalence to their human counterparts thus advocating the dog as a model for future anti-tumour treatment.

## Methods

### Samples

For this study paraffin material of a wide variety of primary liver tumours was available from the paraffin material archive present at the department of Pathobiology, Faculty of Veterinary Medicine, Utrecht University (dog, n = 20), Valuepath, Laboratory for Veterinary Pathology, Hoensbroek, The Netherlands (dog, n = 19), and University Hospitals Leuven, Leuven, Belgium (man, n = 8). In addition, frozen material (dog, n = 7) was available from the tissue bank present at the Department of Clinical Sciences of Companion Animals, Faculty of Veterinary Medicine, Utrecht University. All the material was derived from patients who were submitted for individual diagnostic purposes; no tissue was taken purposely for the reported study. Healthy canine liver samples embedded in paraffin were also available from the Department of Clinical Sciences of Companion Animals, Faculty of Veterinary Medicine, Utrecht University derived from non-liver related research. As a positive control paraffin-embedded liver tissue samples from dogs with fulminant hepatitis and reactive ductular proliferation of HPCs were used (courtesy Dr. J. IJzer, Department of Pathobiology, Faculty of Veterinary Medicine, Utrecht University). All liver tumour samples and fulminant hepatitis samples were fixed in 10% neutral buffered formalin and routinely embedded in paraffin. The paraffin sections (4 μm) were mounted on poly-L lysine coated slides. All the sections (4 μm) were stained with haematoxylin and eosin (HE) for histological determination. To exclude hepatic carcinoids in this study, the following neuro-endocrine differentiation markers were used; chromogranin-A, neuron-specific enolase, and synaptophysin, data not shown [41-43].

### Grading

Histological grading of malignant tumours is based on the grading system of Edmondson and Steiner (ES grading system). The ES grading uses a scale of one to four, with increasing nuclear irregularity, hyperchromatism and nuclear/cytoplasmic ratio, associated with decreasing cytological differentiation for each successively higher grade. The grading system designed for this study is based on the ES differentiation grade and is modified into a four category grading system based on cell morphology (anisocytosis), nuclear morphology (anisokaryosis), presence of multinucleated tumour cells, and mitotic activity.

### Staging

Staging describes the extent or severity of a cancer based on the extent of the original (primary) tumour and the extent of spread in the body. The TNM system is one of the most commonly used staging systems. This system has been accepted by the international union against cancer (UICC) and the American Joint Committee on Cancer (AJCC). The TNM system is based on the extent of the tumour (T), the extent of spread to the lymph nodes (N), and the presence of distant metastasis (M). A number is added to each letter to indicate the size or extent of the tumour and the extent of spread. The staging system used for this study is based on the spread of the tumour through the body, and therefore considered summary staging. Many cancer registries, such as the National Cancer Institutes (NCI) surveillance use summary staging. The staging system used for this study is a modified three category staging system and is based on the invasion and spread of the tumour. The tumours are staged in three categories: Stage 0: macroscopically there is only one tumour process in the liver and/or microscopically the tumour is well circumscribed or encapsulated. There are no indications for intrahepatic or extrahepatic metastases; Stage 1: Microscopically the tumour has spread beyond the original (primary) site to the adjacent tissue and/or vessels or microsatellites can be seen and/or there are macroscopically multiple tumour processes present in the liver; Stage 2: The tumour has spread from the primary site to the lymph node and/or other organs (distant metastasis).

### Immunohistochemistry

Immunohistochemistry (IHC) was performed for K19, K7, HepPar-1, and glypican-3 (GPC-3) on all liver tumour samples. Antibody characteristics, manufacturer, source and dilution are provided in Table 1. Slides were air dried (30 min, RT) and deparaffinised. Heat induced antigen retrieval was performed with 10 mM citrate buffer (pH 6.0) or 10 mM Tris with 1 mM EDTA for 10 minutes in a microwave (850 W) with a cool down period for 10 minutes at RT (Table 3). Antigen retrieval by enzymatic digestion was performed with proteinase K for 15 minutes at room temperature (Table 3). Endogenous peroxidase activity was blocked in 0.3% H_2_O_2 _(30 min) and background staining was blocked with 10% normal goat serum (30 min). The primary antibodies were diluted in the appropriate buffer and incubated as indicated in Table 3. The Envision system was used for secondary antibody labelling (Dakocytomation, Glostrup, Denmark). The signal was developed in 0.06% 3,3'-diaminobenzidine (DAB) solution (Dakocytomation) for 5 minutes and finally counterstained with Mayer's hematoxylin (Mayer's haematoxylin, Klinipath B.V. Duiven, The Netherlands). Negative controls were performed by replacing the primary antibody with washing buffer. Bile-ducts served as an internal positive control for K7 and K19. Hepatocytes from healthy tissue served as a positive control for HepPar-1. Human hepatocellular carcinomas, previously tested to be glypican-3 positive (Department of Morphology and Molecular Pathology, University Hospitals Leuven, Leuven, Belgium) served as a positive control for glypican-3. Staining of human samples for keratin 19, glypican-3, and HepPar-1 were performed as described previously [12,28,44].

**Table 3:** Used antibodies with manufacturer and methods

Antibody	Manufacturer	Type	Clone	Antigen Retrieval	Dilution	Wash Buffer	Incubation
Keratin 19	Novocastra Laboratories Ltd.	mouse monoclonal	B170	Prot K	1:100	TBS	1 hr RT
Keratin 7	Dakocytomation	mouse monoclonal	OV-TL 12/30	Prot K	1:25	TBS	O/N 4°C
HepPar-1	Dakocytomation	mouse monoclonal	OCH 1E5	Tris-EDTA	1:50	PBS	O/N 4°C
Glypican-3	BioMosaics	mouse monoclonal	1G12	Citrate	1:100	PBS	O/N 4°C

Prot K = Proteinase K. RT = Room Temperature. O/N = Over Night.

### Statistics

Two-tailed Fisher's Exact Test was performed to assess associations between keratin 19 positivity and categorical data such as grading, staging, K7 positivity, HepPar-1 positivity, and glypican-3 positivity. Unpaired *t*-test was performed to assess global association between keratin 19 positivity and normally-distributed continuous variable of age. A *P*-value below 0.05 was considered to be significant.

## Competing interests

The authors declare that they have no competing interests.

## Authors' contributions

RS, performed all immunohistochemical stainings, wrote the manuscript and participated in the pathological examination, TI performed the (canine) pathological examination, VD performed the (human) pathological examination, AK performed statistical analysis, LP critically reviewed the manuscript and helped with the study design, JR coordinates the canine tissue bank at the University of Utrecht and helped with the study design, TR devised the study, coordinates the human tissue bank at the University Hospitals of Leuven, and participated in the pathological examination, BS was responsible for the outset of the study and wrote the manuscript. All authors have read and approved the final manuscript.
